# Use of the Consumer Health Literacy Quotient to Quantify and Explore Self-Care Readiness Among Consumers in Four Asia-Pacific Countries

**DOI:** 10.3390/healthcare12222318

**Published:** 2024-11-20

**Authors:** Vandana Garg, Zee Alcasid, Katherine Mendoza, Heesoo Lee, Yi Xin Loo, Andy Nong, Gerard W. Toh, Sheryl Tan

**Affiliations:** 1 Haleon (Formerly GlaxoSmithKline Consumer Healthcare Pte Ltd.), Singapore 139234, Singapore; zee.m.alcasid@haleon.com (Z.A.); katherine.m.mendoza@haleon.com (K.M.); loo.yixin@u.nus.edu (Y.X.L.); sheryl.x.tan@haleon.com (S.T.); 2 School of Biological Sciences, Nanyang Technological University Singapore, Singapore 639798, Singapore; 3 NUS Life Sciences Graduate, Faculty of Science, National University of Singapore, Singapore 637551, Singapore; 4 Toluna, Singapore 048692, Singapore; andy.nong@toluna.com; 5 Tech Observer Asia Pacific, Singapore 318993, Singapore; g.toh@tech-observer.com

**Keywords:** health literacy, self-care, consumer health information

## Abstract

Background/Objectives: Self-care has great potential to benefit consumers and health systems, but its mainstream adoption is hindered by a systemic lack of consumer health literacy (HL). Published data on consumer awareness of self-care and HL are limited for regions in Asia, and are needed to develop interventions to enhance HL and self-care for diverse populations in this region. The aim of this research was to describe and analyze patterns of HL and awareness of self-care among consumers in Asia. Methods: We conducted a mixed-methods study comprising qualitative focus group discussions (FGDs; 64 participants) followed by a quantitative online survey (1200 participants) among consumers in four Asian countries (India, Philippines, Malaysia, and Republic of Korea). We examined five dimensions of HL and self-care relevant to consumers: actively managing health; confidence/skills to find and access health information; confidence/skills to appraise information; support from social circle; and support from healthcare providers. From the quantitative survey, responses for 16 questions covering the five dimensions were used to calculate the Consumer Health Literacy Quotient (CHLQ; normalized range 0–100), an index we developed to assess HL in the context of personal wellness and self-treatable conditions. Latent class analysis (LCA) was applied to identify distinctive patterns of consumer HL within the sample. Results: The mean CHLQ was 75 out of 100 (SD ± 12.9), indicating ‘moderate’ levels of consumer HL across the countries. LCA identified five distinct consumer HL profiles, differing in their average CHLQ (overall score) and along the CHLQ dimensions. Consistent with CHLQ results, the profiles differed in their response patterns for common self-manageable ailments. Conclusions: This study identified distinct patterns of HL and awareness of self-care among consumers in four Asian countries through combined use of the CHLQ and quantitative profiling. This offers a promising approach for understanding self-care-related HL among consumers in Asia. Our findings on patterns of strengths and weakness in specific dimensions of HL and self-care in diverse populations can inform research, communications, and targeted interventions to empower consumers and foster self-care.

## 1. Introduction

Self-care is gaining importance in healthcare today, driven by growing consumer interest in personal health and wellness, as well as by rising medical costs and pressure on national healthcare systems [1]. The importance of consumer autonomy and empowerment in modern healthcare and health promotion is emphasized in the World Health Organization (WHO)’s definition of self-care: *‘the ability of individuals, families and communities to promote health, prevent disease, maintain health, and cope with illness and disability with or without the support of a health worker’* [2].

This shift towards self-care has assumed particular importance for individuals and communities who are constrained by access or affordability of healthcare services [1]. Empowering self-care in populations is crucial to optimize healthcare resources and relieve the strain on primary healthcare services such as hospitals and GPs, and allows individuals to continue their day-to-day activities and maintain their productivity and quality of life [1,3]. Globally, it was estimated that self-care activities have eliminated 11 billion individual hours that would otherwise have been spent on traveling to medical appointments and waiting at medical facilities, and saved about 1.8 billion physician hours that could be better utilized for treating severe medical conditions [1].

In the Asia-Pacific region (APAC), consumer interest in wellness has increased steadily, along with consumer spending on over-the-counter (OTC) medicines and health supplements [4]. Consumers, especially Millennials and Generation Z individuals, increasingly prioritize health and well-being and have a high willingness to pay for product safety and efficacy [5]. This trend has strengthened since the COVID 19 pandemic, with individuals becoming more invested in safeguarding their health, and adopting more preventive measures such as purchasing health and wellness-related products for self-care [5]. Consumers now increasingly consider forms of self-care for conditions such as coughs, colds, and headaches, or visiting their local pharmacies to obtain medication advice, as opposed to visiting a general practitioner (GP) or doing nothing [6].

Formal definitions of self-care, such as the WHO’s, include medical competencies such as disease prevention and self-management for minor health issues that do not require healthcare visits. The self-care continuum therefore encompasses the individual’s health habits and lifestyle, managing self-treatable conditions, and also ‘shared care’ in collaboration with a healthcare provider for managing chronic illnesses and recovery from major trauma [3,7]. However, typical perceptions of self-care are often narrower, revolving around personal wellness activities, healthy eating, exercise, and stress management. Thus, there is enormous potential for greater understanding and adoption of self-care in society, which can enhance health for individuals and improve resource utilization in health systems. Based on a study of 10 countries in different regions worldwide, the Global Self-Care Federation proposed a ‘Self-Care Readiness Index’, which highlights four factors required to build a ‘self-care-ready’ society: support and adoption by stakeholders, consumer and patient empowerment, self-care health policy and the regulatory environment [8].

Studies consistently report low and/or variable health literacy (HL) in populations worldwide, including Asia [9,10,11,12,13,14,15]. Such HL gaps may hinder mainstream adoption of self-care despite its compelling benefits for both individual consumers and societies. For example, instead of factual scientific knowledge, consumers often depend upon tacit knowledge, such as word-of-mouth, brand familiarity, and prior use experience in their self-care decision-making process [16,17]. When searching for health products in-store or making purchase decisions, consumers rely on cues such as brand colors, logos, and graphics as guideposts. Subsequently, when evaluating products to meet specific health needs, consumers are influenced by word-of-mouth from family members and their social circle, and recommendations from healthcare professionals. After successfully using a product, consumers tend to repurchase the same product based on their prior experience when the health need recurs, without considering potentially relevant new information [16,17].

Advances in digital technology for health and medicine and online health resources have made it easier than before for consumers to find information on self-treatable conditions, but the quality and accuracy of this information varies considerably. An online survey of digital HL among university students in China, Malaysia, and the Philippines during the COVID-19 pandemic found that search engines and social media were much more frequently utilized (92.0% and 88.4%, respectively) than websites of doctors or health insurance companies (64.7%), even though the latter were considered trustworthy sources of information [18]. Without strong HL, consumers are less able to appraise the advice they encounter and determine its accuracy and applicability to their own circumstances. Moreover, receiving conflicting information from different sources can create further confusion [6,19]. Such HL gaps have broad implications as they may lead to missed opportunities for timely and accurate action, and incorrect self-diagnosis, potentially hindering effective self-care.

Lastly, it is recognized that interventions to promote HL and self-care should consider the local context (e.g., cultural norms and learning preferences) and be adapted to local needs [20]. Consumer perceptions of self-care may vary across countries and cultures, contributing to the challenges of applying strategies to build ‘self-care-ready’ societies in different regions. For example, national HL policies and resources vary considerably across Asia [21], and there is currently a lack of published research on consumers’ needs and preferences in terms of health education, HL skills, and use of self-care resources within this region. Studies have focused on general HL and adapting existing HL measurement tools for use in Asian populations, or assessing HL in selected groups within individual Asian countries [9,12,13,14,15,22,23,24].

Identifying areas where consumers’ HL is currently adequate or could be improved could help us understand how resources, services, and technology could be designed or adapted to support self-care for a range of consumers within society. Our research aimed to describe and analyze patterns of HL and awareness of self-care among consumers in Asia. We conducted a mixed-methods study in four Asian countries using both qualitative focus group discussions (FGD) and a quantitative online consumer survey. Within the online survey, we incorporated a concise tool that we developed to assess consumers’ HL and capacity for self-care in the context of personal wellness and self-treatable illnesses. This proposed tool, which we refer to as the Consumer Health Literacy Quotient (CHLQ), focuses on specific aspects of HL that may be relevant to consumer health decision-making. Finally, we applied latent class analysis (LCA) modeling [25,26] to these data to identify and explore distinctive patterns of consumer HL within the population. We then profiled each of the identified latent classes in terms of their sociodemographic characteristics and self-care-related attitudes and behaviors. These findings could inform tailored approaches to meet the needs of different consumer segments and provide guidance on adapting communication and engagement strategies for each segment, thus helping to address HL and self-care in more targeted ways.

## 2. Materials and Methods

The study was implemented in two phases: a qualitative phase (focus group discussions), followed by a quantitative phase (online market research survey of consumers). All participants and respondents provided informed consent prior to participation. They were informed that their participation was voluntary, and that their responses would be kept strictly confidential and would be analyzed only in anonymized and aggregated form. The four Asian countries were selected to represent the range that exists across the region in terms of population demographic profiles, ethnic and sociocultural diversity vs. homogeneity, education and human development index levels, and types of health systems. These are all factors thought to influence health literacy and health-seeking behaviors, and thus were considered relevant to our study. The feasibility of recruiting participants given the available research resources and target timeframe were major practical considerations in selecting these four countries.

### 2.1. Qualitative Research (FGD)

Qualitative research via FGDs were conducted in four countries (Malaysia, Philippines, Republic of Korea, and India) to gain a deeper understanding of the current state of consumer HL in the region, and consumers’ perceptions about self-care and its importance in various types of health conditions, including pain relief and immune health. A total of 64 participants took part in small-group FGD sessions within each country. Each session lasted 90 min and involved four participants, led by a professional moderator using a semi-structured discussion guide. The small-group format facilitated active participation and enabled individuals to develop their responses that might not be readily revealed when asked directly in one-on-one interviews [27]. Participants were chosen to be broadly representative of their respective countries in terms of gender, age group, household composition (with or without children), and had to be users of or open to using OTC health products (e.g., multivitamins or mineral supplements, OTC medications). The participants were asked questions on perceptions, attitudes and behaviors related to wellness and self-care, the sources of health information they use, and their confidence in appraising health information. Additionally, questions on special topics of immune health, pain relief, and product information labels were used to stimulate additional discussion on self-care and elicit in-depth responses to corroborate other parts of the discussion.

### 2.2. Quantitative Research (Online Consumer Survey)

In the second phase, a survey questionnaire was developed to validate and extend the key qualitative insights from the FGDs within a larger representative sample of consumers in the same four countries. For greater comparability with the FGD results, the survey questionnaire included similarly worded questions on attitudes and behaviors related to personal wellness and self-care, sources of health information, and confidence in appraising health information. Questions about support from respondents’ healthcare providers and/or social circle, as well as special topics (immune health, pain relief) were also included. The questionnaire was translated into the local language of each country. The online survey was conducted by an independent research agency in accordance with locally applicable codes of conduct for consumer market research. Respondents were recruited from a consumer panel managed by an established global panel provider (https://tolunacorporate.com/product/global-panel-community/ [accessed on 23 Nov 2023]). The panel platform enables anonymous data collection of responses while protecting data privacy. Quota-based filtering was applied to recruit the target sample of 300 respondents per country; this means that, once the target number of survey responses are collected, additional individuals who attempt to complete the online survey are screened out. The filtering criteria applied for the survey were designed to achieve a 1:1 male–female ratio and a sociodemographic profile similar to that of the FGD study, and closely matched the criteria used for FGD study recruitment: respondents aged 18–60 years who purchased or used OTC/non-prescription health products.

### 2.3. Consumer Health Literacy Quotient (CHLQ)

Within the survey questionnaire, we included a set of 16 questions to assess HL in consumer health settings (e.g., use of health supplements or non-prescription medications), using the responses to calculate the CHLQ. Besides functional HL skills of reading and numeracy, additional dimensions such as communicative, interactive, and critical HL are needed to support effective self-care in the context of personal wellness and self-treatable illnesses [28]. The 16 questions for the CHLQ were formulated based on selected questions from the Health Literacy Questionnaire, a self-report-based tool that measures different aspects of the multidimensional construct of HL [29]. Questions were adapted for the current study based on the insights from the FGD phase, and covered five dimensions that we considered particularly relevant to the consumer health setting: (1) actively managing health; (2) confidence and skills to find and access health information; (3) confidence and skills to appraise health information; (4) support from social circle; and (5) support from healthcare providers and the system. Table S1 presents the survey questions used for calculation of the CHLQ. Responses were scored on a 5-point Likert scale (1 = never, 2 = occasionally, 3 = sometimes, 4 = often and 5 = always). The sum of scores for all the responses across the five dimensions (maximum score of 80) was computed and then normalized to the range of 0–100 to form the CHLQ. A higher CHLQ (total normalized score) was interpreted to indicate a higher level of HL and self-care ability (‘high’, 90–100; ‘moderately high’, 79–89; ‘moderate’, 60–78; ‘low’, <60).

### 2.4. Analysis Approach

Survey responses were first analyzed descriptively. Next, a statistical modeling technique, LCA, was conducted on the respondents’ answers to the 16 questions to identify distinct patterns of HL and self-care related attitudes and behaviors. The LCA segmentation technique has been used in social sciences and healthcare studies [30,31,32] to uncover underlying patterns (‘latent classes’) within heterogeneous populations of individuals based on their observed characteristics or responses. Each latent class is taken to represent a comparatively homogenous group of respondents that can be distinguished from other groups within the population. Compared with other methods, LCA offers greater flexibility, as it can utilize a variety of data types (e.g., categorical variables with differing scales) and is able to model classes with differing sizes or other statistical characteristics [25,26]. Additionally, LCA modeling supports the use of statistical model fit criteria to objectively evaluate how well a given model fits the data (number of latent classes and class memberships, i.e., which individuals are assigned to each class). Based on initial analyses to test different segmentation approaches (*k*-means, hierarchical clustering, and LCA), the LCA approach was selected as it generated the most stable and interpretable groupings, with good differentiation of the various segments within the sample.

To determine the number of classes that best represented distinct response patterns (‘consumer health literacy profiles’) within the sample, a series of LCA models with 2–7 classes were constructed and evaluated using model fit metrics. Additional criteria for selection of the ‘best-fit’ model included (1) each class should consist of a minimum of 30 respondents, or at least 5% but not more than 70% of all respondents; (2) each class should exhibit 1–3 discernible patterns in terms of strong or weak scores in specific dimensions. The latent classes in the best-fit model were then profiled in terms of their typical respondent demographics and socioeconomic characteristics, as well as the relevant self-care and HL-related traits that best described each latent class, generating a set of five consumer HL profiles.

## 3. Results

### 3.1. Qualitative Research (FGD)

The FGDs were conducted from July to December 2022 and involved 64 consumers, 16 from each country. Across all four countries, the FGD participants reported that they had a strong motivation to be healthy during the COVID-19 pandemic. Most reported they were able to set goals on health and fitness but struggled to keep up with their health goals post-pandemic as they felt too lazy, tired, or burdened by work. Participants frequently look for and try to validate health information online, using social media channels such as Instagram, YouTube, and Naver (in Republic of Korea), pharmacy websites, WhatsApp channels managed by medical stores, and Wikipedia. Participants expressed confidence in self-managing common conditions such as headaches, coughs, and colds, but were less confident about managing less commonly experienced conditions due to uncertainty about the cause of the illness or symptoms. In such situations, they usually sought advice from a medical professional.

FGD participants from Republic of Korea expressed less confidence in identifying symptoms and were less trusting of digital health sources as they often experienced confusion when faced with conflicting information. Similarly, participants from Malaysia expressed doubts about the accuracy and reliability of online information sources, especially if they could not comprehend the medical/scientific terms used and were unsure how to validate the information presented. When presented with materials on immune health support, participants generally indicated they were familiar with the basic concepts, especially after experiencing the COVID-19 pandemic. Several participants reported that information on certain aspects of the topic (e.g., the variety of different nutrients with roles in immune support) were new and useful to them, and that the interactions prompted them to want to learn more about their current supplements and other wellness products.

### 3.2. Quantitative Research (Online Consumer Survey) and the CHLQ

**Figure 1:**
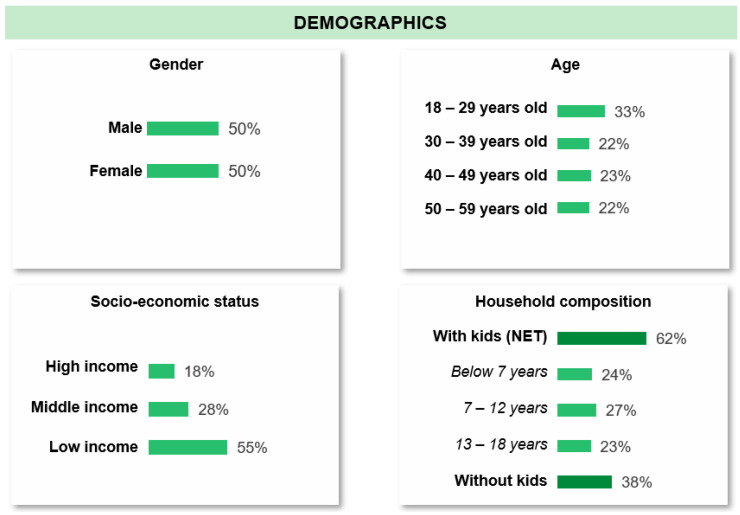
Sociodemographic characteristics of online survey respondents (*n* = 1200).

The online survey was conducted between 15 December 2022 and 9 January 2023 in the same four countries as the FGDs. A total of 1200 consumers from Malaysia, the Philippines, Republic of Korea, and India participated in the online survey. There were 300 respondents from each country with an equal proportion of males and females. The respondents were between 18 and 60 years old (33% between 18–29 years old, 22% between 30–39 years old, 23% between 40–49 years old, and 22% between 50–59 years old) (Figure 1). More than half of the respondents (55%) had low income, 28% had middle income, and 18% had high income (relative to income levels in their respective countries); and most respondents (62%) had children.

For the overall sample of 1200 respondents, the mean CHLQ (total normalized score) was 75 out of 100 (SD ± 12.9) but ranged from 66.4 (SD ± 13.4) in Republic of Korea to 78.6 (SD ± 10.8) in the Philippines. The mean CHLQ for Malaysian respondents was 76.7 (SD ± 10.8) and 78.4 (SD ± 12.3) for respondents in India. This range was taken to correspond with generally ‘moderate’ levels of consumer HL across the four countries.

### 3.3. Identification of Distinct Consumer Health Literacy Profiles

**Figure 2:**
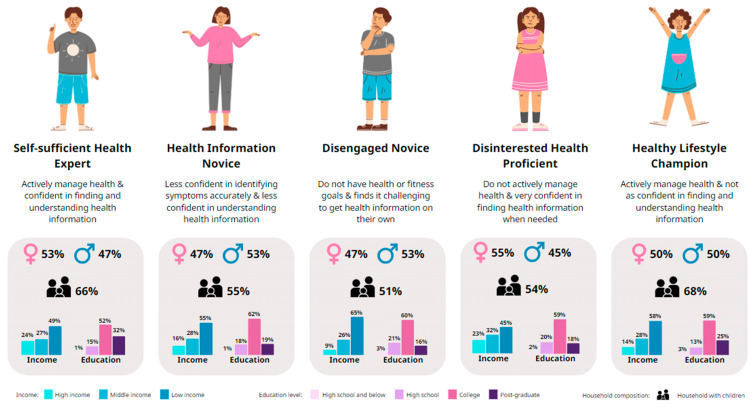
Five distinct consumer health literacy profiles identified through latent class analysis.

From a series of LCA models with 2–7 latent classes, a model with five classes was selected based on statistical model fit criteria as well as differentiation and interpretability of the classes identified. We then proposed the following five descriptors for these consumer HL profiles, ‘Self-sufficient Health Expert’, ‘Health Information Novice’, ‘Disengaged Novice’, ‘Disinterested Health Proficient’, and ‘Healthy Lifestyle Champion’ (Figure 2).

These descriptors aim to capture the different patterns of HL among respondents, based on their CHLQ (overall) and domain sub-scores, as well as their associated behavioral traits (Table 1).

The number of respondents classified into each class ranged from 93 (8% of the overall survey sample) to 416 (34% of the overall sample). Table 2 shows the demographic and socioeconomic characteristics of each class. The distributions of age group and gender were generally similar across the five classes, with some variation across the classes in terms of household composition, employment status, income, and education level.

Based on their CHLQ, the ‘Self-sufficient Health Expert’ (90.7), ‘Health Information Novice’ (64.0), and ‘Disengaged Novice’ (48.7) classes were well differentiated (Table 3).

**Figure 3:**
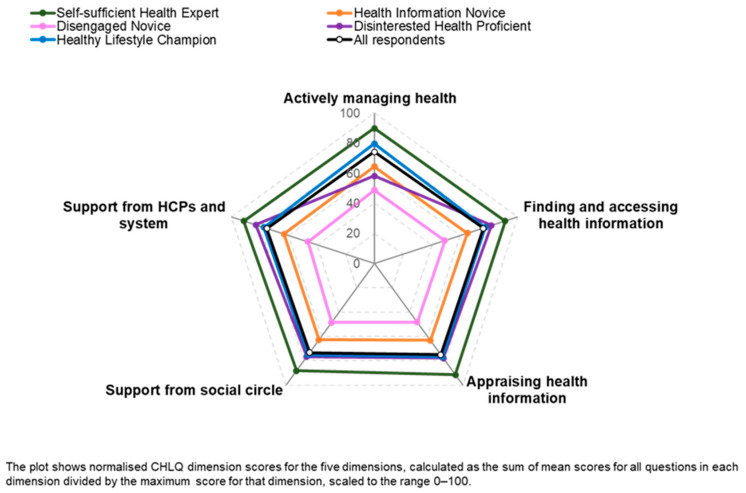
Consumer Health Literacy Quotient (CHLQ) domain scores for the five consumer health literacy profiles.

The remaining two classes, ‘Disinterested Health Proficient’ (74.7) and ‘Healthy Lifestyle Champion’ (77.6), had similar CHLQ (normalized total scores) but could be differentiated based on their responses in the ‘Actively managing health’ domain. Figure 3 shows the patterns of responses (represented by mean sub-scores) across the dimensions for each of the five classes. The ‘Self-sufficient Health Expert’ class had the highest sub-scores for all five dimensions, followed by ‘Healthy Lifestyle Champions’, ‘Health Information Novices’, and ‘Disengaged Novices’. Across four of the five dimensions, sub-scores for each class were generally distinct and consistently different from those of other classes. The exception was the ‘Disinterested Health Proficient’ class, which had a slightly lower score in terms of ‘Actively managing health’ but was similar to the ‘Healthy Lifestyle Champion’ class across the other four dimensions (Figure 3).

### 3.4. Consumer Health Literacy Profiles

‘Self-sufficient Health Experts’ represented a quarter (25%) of the overall sample. With the highest CHLQ (mean: 90.7) and the highest domain sub-scores of all the classes, ‘Self-sufficient Health Experts’ also had the highest levels of HL and confidence in self-care. They are both health conscious and fully confident in their ability to search for health information and synthesize information to manage their health, including self-treatable conditions. They also receive strong support from their social circle and have access to a reliable healthcare provider.

‘Health Information Novices’ represented another quarter (25%) of the survey respondents. With a mean CHLQ of 64.0, ‘Health Information Novices’ had intermediate levels of HL and confidence in self-care. They are somewhat health conscious (e.g., setting personal health goals and leading an active lifestyle), but are less confident in finding and processing health information independently. They also do not have trusted healthcare providers that they would consult for medical advice. 

The ‘Disengaged Novice’ class included 8% of the overall survey sample. With the lowest CHLQ (mean total score: 48.7), this class also had the lowest sub-scores in all dimensions. ‘Disengaged Novices’ had the least adequate HL and may be the least adept at self-care. They appear relatively unconcerned about their health and do not take active steps to manage it. They are also not confident in using health information to manage their health or self-treatable conditions. They have little support from their social circle and do not have a reliable healthcare provider to go to for medical advice. 

The ‘Disinterested Health Proficient’ class included 8% of the overall sample. With a moderately high CHLQ of 74.7, this class had relatively high domain sub-scores except for ‘Actively managing health’. ‘Disinterested Health Proficient’ respondents had a moderately high level of HL and confidence in self-care. Although not actively engaged in managing their health, they are confident about finding the right health information when needed and generally feel able to manage self-treatable conditions. They can access healthcare support and medical advice from trusted healthcare providers when needed. 

‘Healthy Lifestyle Champions’ were the largest class identified, representing 34% of the overall sample. With a mean CHLQ of 77.6 (the second highest among the five classes), they had moderately high levels of HL and confidence in self-care. Notably, although they had a similar mean CHLQ to that of the ‘Disinterested Health Proficient’ class, ‘Healthy Lifestyle Champions’ showed a stronger interest in taking care of their health, mainly through diet and lifestyle, and adopted various measures to prevent illness. They reported confidence in managing self-treatable conditions, as well as good support from both their social circle and healthcare providers.

**Figure 4:**
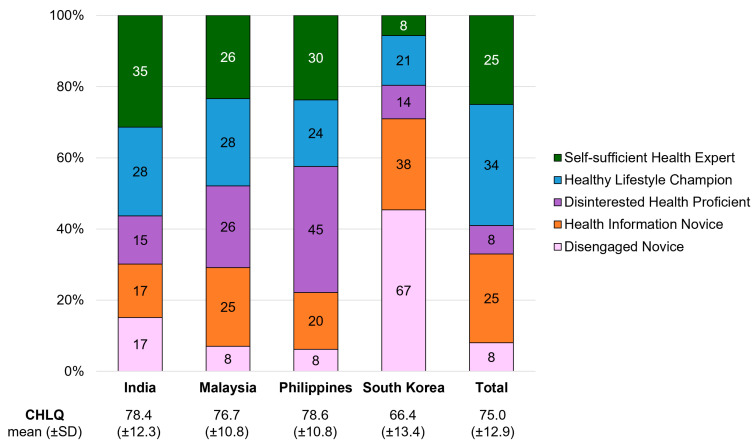
Distribution of consumer health literacy profiles within each country.

Among the four country samples, the Republic of Korea sample had the highest proportion (67%) of ‘Disengaged Novices’, whereas the Philippines and Malaysia had the smallest (8% for both countries). The Republic of Korea sample also contained the smallest proportion (8%) of ‘Self-sufficient Health Experts’ (Figure 4). These country differences in segment distribution may be major drivers of the variation in mean CHLQ scores across the countries.

**Figure 5:**
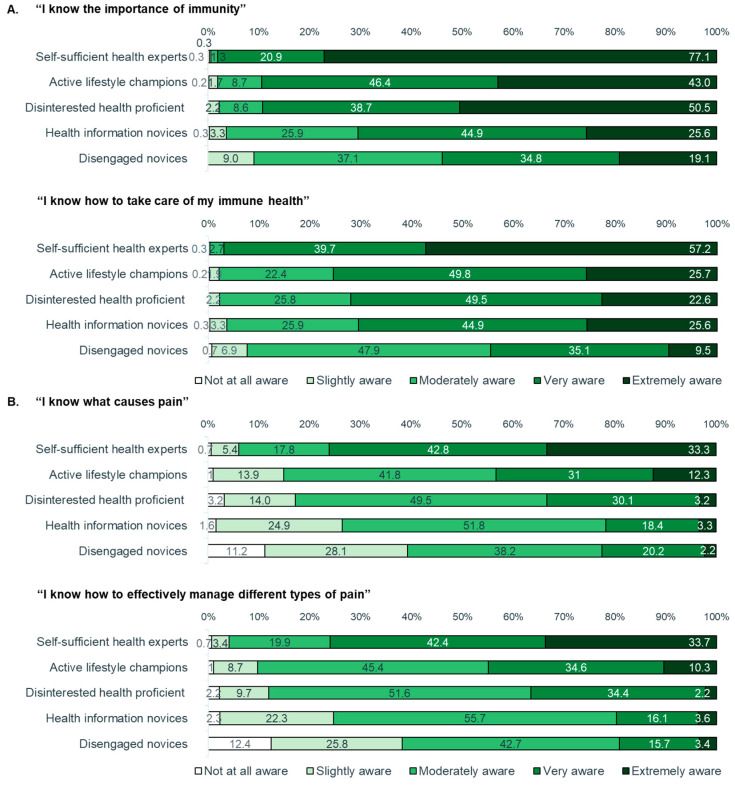
Responses to questions on (**A**) immune health and (**B**) pain relief for the five consumer health literacy profiles.

Finally, we explored whether these variations across segments in terms of CHLQ and HL profiles were consistent with those observed for two other common consumer health topics. Specifically, we examined how the five segments differed in terms of their attitudes and perceived awareness about self-care for immune health and for pain. Of the two topics, respondents were more confident about their awareness of immune health and how to take care of it than about the causes and management of different types of pain (overall median rating of 4, ‘very aware’ for immune health; overall median rating of 3, ‘moderately aware’, for managing pain) (Figure 5A,B). Notably, this trend was consistent across all segments. For both immune health and pain relief, we consistently observed that the segments with lower CHLQ were less confident than those with higher CHLQ about their awareness and ability to self-manage in these areas. For example, the ‘Disengaged Novices’ and ‘Health Information Novices’ tended to rate their awareness of what causes pain and how to manage different types of pain somewhat lower compared with the other three groups (Figure 5B).

## 4. Discussion

This study yielded novel qualitative and quantitative insights on the HL and self-care-related characteristics of consumers in four countries across the Asia region. The qualitative research (FGD) results revealed that, although many consumers are aware of the importance of health and are interested in learning to take care of their health, they may feel that they do not possess sufficient knowledge, confidence, and/or skill to do so independently. The FGD results also suggested that, among consumers in Asian countries, the prevailing understanding of self-care is focused on having a ‘healthy lifestyle’ (diet and physical activity), potentially overlooking other aspects of the self-care continuum, such as self-management of minor health issues that do not require physician visits.

Results from the quantitative survey illustrate the potential use of a consumer-specific HL tool combined with segment profiling to assess multiple dimensions of attitudinal and behavioral patterns that are relevant to self-care. The online consumer survey, conducted in the same countries as the FGD, indicated similar trends of moderate awareness and interest in health matters, while revealing certain areas where consumers felt less knowledgeable and confident. Using responses to a set of 16 HL-related questions, we derived and used the CHLQ to quantify respondents’ level of HL and self-care readiness in the context of everyday health and wellness activities. Similar to existing tools that assess one or more specific HL domains (e.g., disease prevention, navigation of the healthcare system) [10,33,34], the CHLQ represents a simple score to capture aspects of HL relevant to consumer health and self-care. The mean CHLQ was 75 (out of 100) for the overall sample and ranged from 60–78 for individual countries. These scores indicate moderate but not notably high levels of HL and confidence in self-care ability in this sample.

The observed variations in CHLQ between and within countries prompted further exploration and segment profiling using LCA. This analysis enabled five distinct consumer HL profiles to be identified within this sample, which we characterized as: ‘Self-sufficient Health Expert’, ‘Health Information Novice’, ‘Disengaged Novice’, ‘Disinterested Health Proficient’, and ‘Healthy Lifestyle Champion’. The LCA approach has been used in studies of health risks and health-related behaviors to identify meaningful and actionable population subgroups or segments [35,36,37,38]. Here, applying quantitative profiling to health-related attitudes and behaviors adds a novel perspective by revealing diverse ways that individuals engage with and take action to maintain their health. The findings also indicate that the HL dimensions included in the CHLQ are indeed relevant in consumer health settings. The combined approach described here may improve recognition and targeting of those with needs in more than one dimension. For those who report both limited awareness and low levels of engagement with health matters, raising awareness of health status or risks could potentially also increase willingness to practice self-care.

We note that the five segments identified here typify recognizable attitudinal–behavioral profiles in terms of personal HL and confidence in self-care. For example, a recent Global Self-Care Federation report described two contrasting literature-based personas, representing individuals with ‘high’ versus ‘limited’ self-care readiness, to illustrate the different types of opportunities and challenges that individuals may face in their self-care journey. Our quantitative analysis of inter-individual variation among consumers adds to earlier qualitative descriptions, and reinforces that initiatives for strengthening HL and self-care should be optimized according to the distinct needs and challenges of individuals [39].

Specifically, combining the CHLQ and data-driven profiling analysis enabled a more nuanced understanding of HL needs among consumers. To further assess the applicability of the approach, the five segments’ response patterns were compared for two common self-care topics: immune health and pain relief. Consistent with the CHLQ results, the ‘proficient’ segments tended to report greater topic-specific awareness and confidence in self-management, especially the Self-sufficient Health Experts, compared with the Disengaged Novices and Health Information Novices. These observations further support the relevance of the CHLQ as a measure in common consumer health contexts, such as understanding and using OTC products for immune health or pain relief.

Based on the CHLQ and the response patterns, a third of the respondents could be described as ‘novices’ (Health Information Novices and Disengaged Novices), with lower awareness and confidence in various aspects of self-care and wellness. This observation is consistent with reports of variable and/or inadequate HL in Asian populations, emphasizing the need for educational initiatives on healthcare, disease prevention and health promotion for the general public [3,8,11,12,13,14]. Where population studies of general health literacy have been undertaken within Asia, the broad trends appear similar to those for Europe or North America [9,40,41]. In addition to high rates of limited HL reported in both Western and Asian populations, similar trends identified were the associations of higher education attainment and social status with greater health literacy (termed the ‘social gradient’ of HL), and differential distribution of HL levels in sub-populations [9,41]. Consistent with these findings, studies in Southeast Asian countries have reported that educational attainment, age, income, and socioeconomic background were the most common factors associated with limited HL [11]. In our analysis, age and gender distributions were broadly similar across the five segments, suggesting that factors other than gender or age, such as the regional or cultural context, could also be related to health-related attitudes and behaviors, thus warranting further studies.

Another notable trend is the increasing focus on digital HL or eHealth literacy, complementing other forms of HL interventions [42,43]. Digital HL has assumed even greater importance with the availability of generative artificial intelligence (GAI) tools such as ChatGPT, which was first made available to the public in November 2022. Although GAI offers exciting possibilities for improving access to health information (availability on-demand 24/7, in multiple languages, potential for personalization and interactivity), issues such as accuracy, reproducibility, bias, and privacy need to be properly addressed [44,45]. The implications of these issues have been highlighted in recent studies [44,46,47], emphasizing that digital HL is perhaps even more important now than before.

Beyond enriching the current understanding of self-care-related attitudes, abilities, and behaviors of Asian consumers, insights from such healthcare consumer profiles may assist health policy makers and advocacy organizations in determining priorities for self-care education. These insights may also guide customized interventions to increase the adoption of self-care among consumers. Across both FGDs and the online survey, one recurring need articulated by consumers was for trusted resources to help them validate and understand seemingly conflicting information on health topics from multiple sources, such as social media channels, blogs or online health forums. This reinforces existing research on the importance of HL in increasing consumers’ ability to evaluate online health information and their levels of trust in online health information [48].

Given the complex multidimensional nature of HL, one of the strengths of combining CHLQ and segment profiling is that it offers a more comprehensive way to capture the self-care readiness of individuals and groups, and infer the types of support they may require in health-related interactions (e.g., point-of-sale or health service touchpoints). Other potential dimensions relevant to self-care by consumers, such health-related ‘activation’ [49], could be explored in future work.

One potential limitation is that the current CHLQ is a subjective, self-report-based measure, and does not measure HL performance or skill (e.g., knowledge or numeracy). However, a systematic review in diabetes management showed that both subjective and objective assessments of HL are relevant to understanding health outcomes: diabetes self-care was best predicted by self-report measures, whereas knowledge was best predicted by objective measures [50]. In this study, LCA was applied to the overall cross-sectional survey sample, which included respondents from four different countries. The variation in mean CHLQ across countries appears to be reasonably well explained by the respective proportions of the five segments in each country; however, further research is needed to contextualize the findings with respect to available HL data for these countries [12,13,14], owing to the differences in study populations and methodology. Also, since these data are cross-sectional and included only four countries, further validation in other datasets will be needed to ascertain the robustness of the findings and the generalizability of the approach across different consumer populations. An expanded dataset would also support country comparisons, especially if complemented by a deeper understanding of the specific local sociocultural context of self-care [51]. This could generate further meaningful insights and guide development of suitable localized strategies; for example, focusing on large or ‘actionable’ subgroup(s) within a population such as the health ‘novice’ segments described in our analysis. We note that, while such insights can aid in designing interventions to enhance the awareness and capabilities of individuals/groups, they can also be applied to designing services and technology to better serve the needs of diverse groups.

## 5. Conclusions

This mixed-methods study identified distinct patterns of HL and awareness of self-care among consumers in four Asian countries, through quantitative profiling based on the dimensions captured by the CHLQ tool. The findings on distinct self-care-related attitudes and behaviors across groups of consumers suggest this may be a promising approach for tailoring health information delivery and engagement according to individuals’ levels of self-care-related HL in Asia. The findings could inform and support the development of improved strategies by national organizations, stakeholder groups, and industry to achieve greater consumer empowerment and foster a culture of self-care.

## Figures and Tables

**Table 1:** Self-care-related characteristics of the five consumer health literacy profiles.

Profile	Self-Sufficient Health Expert *n* = 297 (25%)	Health Information Novice *n* = 305 (25%)	Disengaged Novice *n* = 89 (8%)	Disinterested Health Proficient *n* = 93 (8%)	Healthy Lifestyle Champion *n* = 416 (34%)
Description	They are highly health conscious and have full confidence in their ability to search for health information and synthesize the information they find to manage their own health. They have strong support from healthcare providers and the system.	Although they are slightly more conscious about their health (leading an active lifestyle, setting their own goals on fitness), they are less confident in finding and processing health information on their own, which may translate to being less competent in self-managing health.	They are not concerned with health matters, not actively managing their health and have limited knowledge and understanding of health information. They also do not have strong support from healthcare providers and the system.	They are not actively managing their health but are very confident in their ability to find and process health information. However, when they are presented with differing information, they may become confused.	They take care of their health by having a balanced diet and an active lifestyle. When it comes to health, they tend to be more preventive than reactive. They are confident in managing their health and conditions which are self-treatable.
Behavior traits	Actively manage health (especially setting their own goals on health and fitness; making efforts to be healthy).	Less confident in identifying symptoms accurately and following health advice.	Do not have goals on health and fitness.	Do not actively manage their health.	Actively manage health (especially setting their own goals on health and fitness; making efforts to be healthy) Strong focus on balanced diet and preventing illness.
	Confident in finding and understanding information from a variety of sources, even if the information appears different.	Do not really know where to find reliable and accurate health information. Less confident in understanding different information about the same health topic from multiple sources.	Unable to obtain health information by themselves. May become confused when they receive different information about the same health topic from multiple sources. Find it challenging to obtain health information in words they understand.	Very confident of finding and accessing information accurately and when they need it.	However, they may be less confident about understanding different information on a health topic from multiple sources.
	Confident in using health information to manage their own health, including conditions that can be self-managed.	Less confident in using health information to manage their own health and self-treatable conditions.	Less confident in using health information to manage their own health and self-treatable conditions. Less confident about understanding and following medicine pack instructions.	Confident about processing health information and information about medicines.	Confident in using health information to manage their own health and self-treatable conditions.
	Able to access the right healthcare support whenever they need to.	Do not have trusted healthcare providers to go to for advice or seek treatment.	Do not have trusted healthcare providers to go to for advice or seek treatment.	Able to access healthcare support when needed, and have moderately high trust in healthcare providers and the system.	Generally have trusted healthcare providers to go to for advice or treatment.

**Table 2:** Sociodemographic characteristics of the five consumer health literacy profiles.

	Total	Self-Sufficient Health Expert	Health Information Novice	Disengaged Novice	Disinterested Health Proficient	Healthy Lifestyle Champion
*n* of individuals	*n* = 1200	*n* = 297	*n* = 305	*n* = 89	*n* = 93	*n* = 416
Segment size	100%	25%	25%	8%	8%	34%
Age group (years)						
18–29	33%	32%	34%	30%	27%	34%
30–39	22%	27%	22%	19%	23%	20%
40–49	23%	22%	22%	25%	22%	23%
50–59	22%	19%	22%	26%	29%	23%
Gender						
Male	50%	47%	53%	53%	45%	50%
Female	50%	53%	47%	47%	55%	50%
Household composition						
With children	62%	66%	55%	51%	54%	68%
Without children	38%	34%	45%	49%	46%	32%
Employment status						
Full-time student	16%	12%	19%	13%	19%	15%
Part-time student	1%	0%	1%	2%	2%	1%
Full-time employed	67%	77%	61%	57%	58%	69%
Part-time employed	8%	5%	11%	13%	5%	6%
Not employed	3%	1%	3%	3%	6%	3%
Retired	1%	1%	1%	1%	1%	1%
Homemaker/housewife	5%	3%	4%	9%	8%	5%
Income level						
High income	18%	24%	16%	9%	23%	14%
Middle income	28%	27%	28%	26%	32%	28%
Low income	55%	49%	55%	65%	45%	58%
Education level						
Some high school or below	2%	1%	1%	3%	2%	3%
High school	16%	15%	18%	21%	20%	13%
College	59%	52%	62%	60%	59%	59%
Postgraduate	23%	32%	19%	16%	18%	25%

Income level categories were defined as follows: India: low income, INR 5–15 Lakh rupees; middle income, INR 15–20 Lakh rupees; high income, >INR 20 Lakh rupees. Malaysia: low income, RM 36,000–59,999; middle income, RM 60,000–83,999; high income, >RM 84,000. Philippines: low income, PHP 200,000–499,999; middle income, PHP 500,000–999,999; high income, ≥PHP 1,000,000. Republic of Korea: low income, 30,000,000–70,000,000 KRW; middle income, 70,000,000–100,000,000 KRW; high income, ≥100,000,000 KRW.

**Table 3:** Consumer Health Literacy Quotient (CHLQ) and domain sub-scores of five consumer health literacy profiles.

3	Total	Self-Sufficient Health Expert	Health Information Novice	Disengaged Novice	Disinterested Health Proficient	Healthy Lifestyle Champion
*n* of individuals	*n* = 1200	*n* = 297	*n* = 305	*n* = 89	*n* = 93	*n* = 416
Segment size	100%	25%	25%	8%	8%	34%
Total score (range: 0–100)	75.0	90.7	64.0	48.7	74.7	77.6
1. Actively managing health (four questions)	74.3	89.7	64.7	49.0	59.1	79.5
2. Confidence and skills to find and access health information (four questions)	76.0	91.2	65.0	49.4	81.4	77.6
3. Confidence and skills to appraise health information (five questions)	75.0	91.4	63.2	48.7	78.0	76.7
4. Support from social circle (one question)	73.4	88.0	63.1	48.9	75.9	75.0
5. Support from healthcare providers and the system (two questions)	75.3	91.0	63.2	47.0	83.3	77.1

## Data Availability

The datasets analyzed in this article are proprietary and not publicly available. Questions about the datasets should be directed to vandana.x.garg@haleon.com.

## Supplementary Materials

The following supporting information can be downloaded at: https://www.mdpi.com/article/10.3390/healthcare12222318/s1, Table S1: Survey questions used for calculation of the Consumer Health Literacy Quotient (CHLQ).
