# Supplementary material for: Use of the Consumer Health Literacy Quotient to Quantify and Explore Self-Care Readiness Among Consumers in Four Asia-Pacific Countries
Source: Healthcare (Basel). 2024 Nov 20;12(22):2318. doi: 10.3390/healthcare12222318 (PMC11593463; doi:10.3390/healthcare12222318)
Supplement: Supplementary file 1 [file healthcare-12-02318-s001.zip › healthcare-3268827-supplementary.pdf]

SUPPLEMENTARY MATERIAL

Supplementary Table S1. Survey questions used for calculation of the Consumer Health Literacy Quotient (CHLQ)

	Always	Often	Sometimes	Occasionally	Never
**Actively Managing Health**<br>1. I set my own goals about health and fitness.<br>2. There are things that I do regularly to be healthy.<br>3. I eat a balanced, healthy diet.<br>4. I engage in actions to prevent illness.					
**Confidence and skills to find and access information**<br>5. I know where to find reliable and accurate health information.<br>6. I am able to find information about health and health problems when I need it.<br>7. I am able to get health information in words I understand.<br>8. I am able to get health information by myself.					
**Confidence and skills to appraise health information**<br>9. I am able to accurately identify symptoms and follow health advice.<br>10. I am able to understand health information.<br>11. I have clear understanding even if different sources have different information about the same health topic.<br>12. I feel confident using the health information to manage my own health and self-manageable conditions<br>13. I feel confident to understand and follow the instructions on pack.					
**Support from social circle**<br>14. I have family and friends who can give me useful advice on health so that I can confidently self-manage my health.					
**Support from healthcare providers and system**<br>15. I am able to access the right healthcare support whenever I need to (e.g., clinics, hospitals).<br>16. I have at least one healthcare provider who I can trust and go to for advice/seek treatment (e.g., doctors, pharmacists).					
